# Death of Dr. Chas. A. Jourdan from Effects of Chloroform

**Published:** 1877-05

**Authors:** 


### ARTICLE II.

### *Death of Dr. Charles A. Jourdan from the Direct Eff cts of Chloroform.*

At half-past four o'clock P. M., Dr. Jourdan was put under the influence of chloroform for the purpose of having the left eye extirpated, which had received an injury five months previously, while he was living in Texas.

The patient was placed on the operating table, and chloroform was administered in the usual way, which soon brought on that sleep that knew no wakening; the eye was speedily removed when the pulse and breath simultaneously ceased and all efforts to resuscitate were unavailing; he never breathed after the first alarming symptoms were noticed. He had a good constitution and no organic disease of any kind, which was proven by post mortem examination.

Dr. Jourdan was born and raised in East Tennessee, and practiced dentistry many years in Athens, Tennessee; but for the last ten years had practiced in Huntsville, Alabama, until twelve months ago, when he removed to Terrell, Texas, where he practiced until he received the accident above referred to.

Dr. Jourdan was a true gentleman and an honest man; as an operator he was among the first; as a friend he was true and noble; to know him was to like him. Society and the profession have sustained a great loss, and his family an irreparable bereavement.

S. P. C., Memphis, Tenn.,
April 7th, 1877.

---

### ARTICLE III.

*The Susquehanna Dental Association.*

